# Dermoscopy of Basal Cell Carcinoma Part 2: Dermoscopic Findings by Lesion Subtype, Location, Age of Onset, Size and Patient Phototype

**DOI:** 10.3390/cancers17020176

**Published:** 2025-01-08

**Authors:** Irena Wojtowicz, Magdalena Żychowska

**Affiliations:** Department of Dermatology, Institute of Medical Sciences, Medical College, Rzeszow University, 35-310 Rzeszow, Poland

**Keywords:** dermoscopy, dermatoscopy, basal cell carcinoma, BCC subtypes, nodular BCC, superficial BCC, high-risk BCC

## Abstract

**Introduction:** Basal cell carcinoma (BCC) is the most prevalent type of skin cancer worldwide. Despite its low metastatic potential, certain subtypes present an aggressive clinical course. Part II focuses on the different dermoscopic patterns observed in BCC, depending on the lesion subtype, its location on the body, the patient’s age, the size of the tumor, and skin phototype. **Methods**: A search of the PubMed database was conducted for studies reporting dermoscopic findings in BCC across all body locations, histopathologic subtypes, tumor sizes, ages of onset and skin phototypes. **Results:** There are no dermoscopic features indicative of a particular BCC subtype. However, arborizing, truncated or glomerular vessels, shiny white lines, ulceration, white areas, absence of pink zones and large blue-gray ovoid nests suggest high-risk BCCs (morpheaform, micronodular, infiltrative, basosquamous). Pigmented features can occur in all BCC types, though increased pigmentation indicates less aggressive subtypes (nodular, superficial, fibroepithelioma of Pinkus, adenoid). BCCs most commonly develop on the head, typically presenting as nodular and non-pigmented tumors. Those on the nose, eyes and ears may be more aggressive and prone to recurrence. On the trunk, BCCs are usually superficial and pigmented. Lower limb lesions often show polymorphous vessels rather than arborizing ones, which makes the dermoscopic diagnosis challenging. Dermoscopy aids early detection, with larger tumors exhibiting more established features but no size-specific patterns. Aggressive subtypes display similar dermoscopic findings regardless of size. **Conclusions:** Dermoscopy is a valuable tool for the early detection of BCC, though no specific dermoscopic features can definitively identify subtypes. High-risk BCCs can be suspected when distinct vascular and structural patterns are present, particularly in lesions located on the face, especially around the nose, eyes and ears, while pigmented features may indicate less aggressive subtypes.

## Simple Summary

Basal cell carcinoma (BCC) is the most common form of skin cancer with different levels of aggressiveness depending on the subtype. High-risk BCCs can be suspected when the correlation of certain vascular and structural features occurs, especially in areas like the nose, eyes and ears. On the other hand, pigmented features have been found to be more common in less aggressive subtypes. Dermoscopy, a non-invasive diagnostic tool, improves early detection of BCC and helps in determining the subtype. Nevertheless, dermoscopic challenges remain, particularly in the case of lesions located on the lower limbs.

## 1. Introduction

Basal cell carcinoma (BCC) is the most common skin cancer, with incidence rising globally [1,2,3]. Although typically slow-growing and rarely metastatic, certain subtypes are more aggressive, increasing the risk of recurrence and morbidity [4]. Early and precise diagnosis is crucial for effective management. Dermoscopy, a non-invasive tool, helps clinicians identify specific patterns and has been proven to play crucial role in the initial detection of BCC [1,4,5,6]. Part 2 of the review summarizes the prevalence of the dermoscopic findings in BCC with particular emphasis on variations depending on lesion subtype, location, age of onset, size and patient phototype.

## 2. Methods

**Figure 1:**
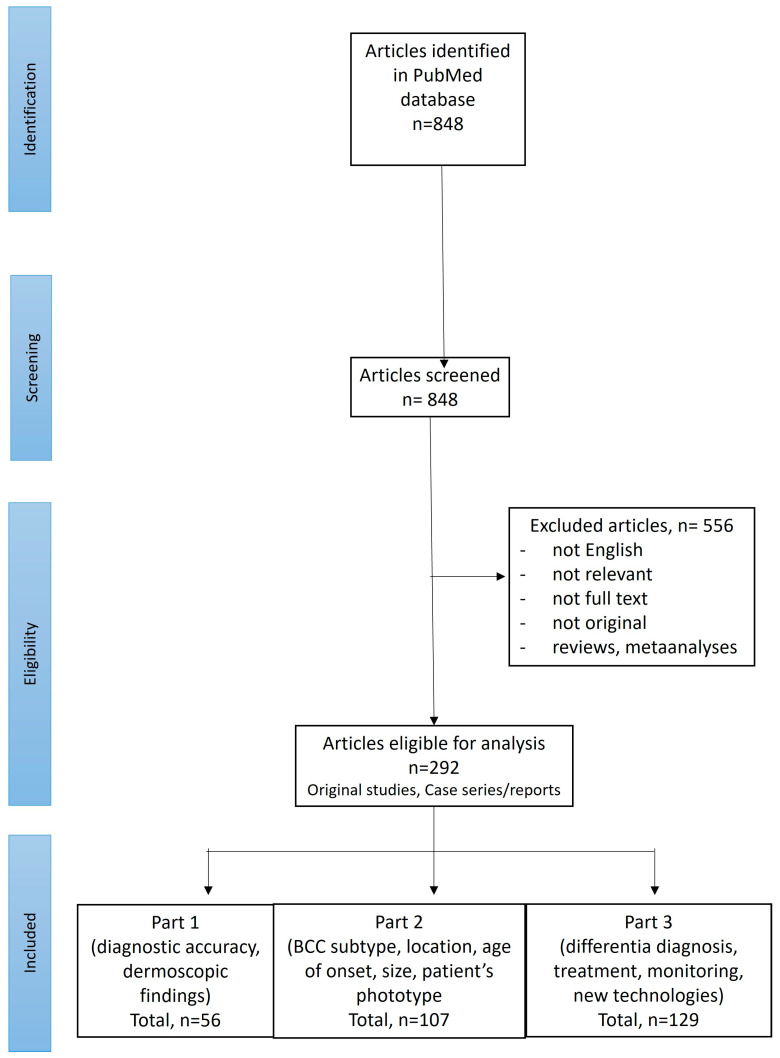
PRISMA flow chart illustrating the screening procedure.

A search of PubMed was performed for English-language publications using the following search term: “(BCC OR basal cell carcinoma OR basalioma) AND (dermoscopy OR dermatoscopy)”. Records available from the inception of the PubMed database until September 2024 were screened. The references of the initially identified papers were also checked. Two reviewers (I.W. and M.Ż.) performed the screening of the abstracts and, if considered relevant, the full texts were subsequently reviewed. Only original studies or cases reporting dermoscopic features in histopathologically confirmed BCCs (located anywhere on the body, of any histopathologic subtype, size and at any age of onset) were included. Figure 1 illustrates the selection process of the articles according to the PRISMA (Preferred Reporting Items for Systematic Reviews and Meta-Analyses) standard.

## 3. Results

Out of 848 studies initially identified in the literature search, 292 were selected for further review. Of these, 107 articles discussed dermoscopic findings in BCCs depending on the lesion subtype, location, age of onset, size or patient phototype and are included in Part 2 of the review.

### 3.1. Dermoscopic Findings by BCC Subtype

The literature search revealed 52 studies that focused on the dermoscopic features of various BCC subtypes.

More than 26 different variants of BCC have been distinguished in the literature [7]. Some subtypes exhibit more aggressive behavior, with increased morbidity and recurrence risk. These “high-risk” or “aggressive” subtypes, comprising nearly 5% of all BCCs, include sclerodermiform (also known as morpheaform, morphoeic or sclerosing), micronodular, infiltrative, and basosquamous BCCs. In contrast, “low-risk” or “non-aggressive” subtypes include nodular, superficial, fibroepithelioma of Pinkus and adenoid BCCs [8,9,10,11,12,13,14,15,16,17,18,19].

No specific dermoscopic structures have been found to be indicative of a particular BCC subtype [9,11]. Furthermore, studies show that dermoscopic findings are rather correlated with tumor thickness than with subtype [9,13,20]. Negrutiu et al. reported that the depth of invasion index was directly related to the presence of arborizing vessels and ulceration, but negatively correlated with short, fine telangiectasias, maple-leaf-like areas and spoke-wheel areas [20].

Verduzco-Martínez et al. identified arborizing and truncated (short, linear path, diameter of 0.01–0.02 mm, abrupt interruption) vessels as highly specific for diagnosing high-risk BCCs. The authors also noted that ulceration should raise suspicion of an aggressive subtype [17]. Other studies confirmed that arborizing vessels should be considered the most significant feature indicative of aggressive BCCs [13,21]. Pyne et al. found that aggressive BCCs often lack pink areas or they constitute less than half of the tumor area [16]. Kim et al. created a “dermatoscopic index of BCC aggressiveness”, assigning “+1” for multiple blue-gray globules, arborizing telangiectasia and concentric structures and “−1” for large blue-gray ovoid nests. A score over “+2” indicated aggressive BCCs [14].

#### 3.1.1. Sclerodermiform BCC

Dermoscopic features of sclerodermiform BCC include arborizing vessels or microvessels on a milky red, pink-white or porcelain-white background, along with multiple erosions or ulcerations [9,10,15,18,22]. Arborizing vessels and ulcerations were found to be more common in the head and neck than on the body [22]. Diagnosis is often delayed due to the deep invasive nature of this variant. Typically, the tumor needs to reach a larger size before the arborizing microvessels can be seen under dermoscopy [23]. Ulcerations can form a ring, as noted by Inamura [24]. Sclerodermiform BCC typically has poorly defined margins and is non-pigmented [10,22].

#### 3.1.2. Micronodular BCC

**Figure 2:**
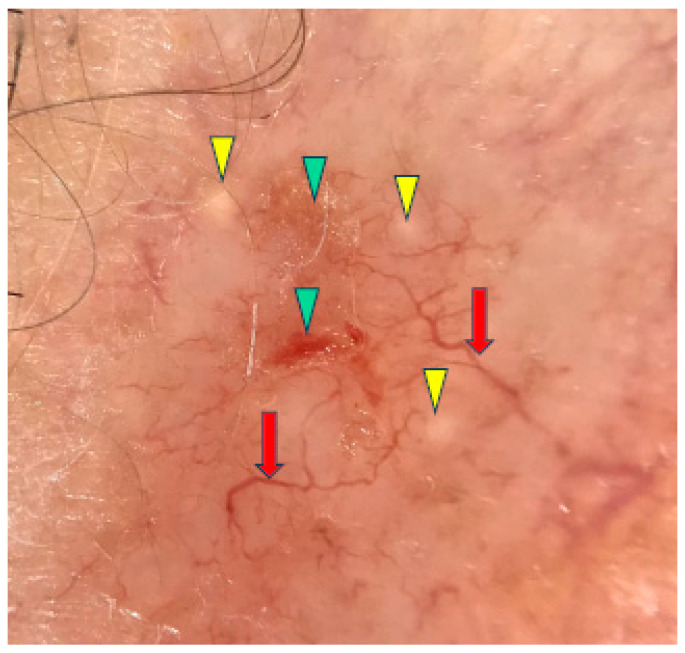
Dermoscopy image of micronodular BCC showing arborizing vessels (red arrows), erosions (green arrowheads), milia-like cyst (yellow arrowheads).

Under dermoscopy, the micronodular subtype of BCC was frequently pigmented with brown globules and blue globules/nests. Common dermoscopic findings also included short fine telangiectasias, arborizing vessels, milky red structureless areas, ulceration and white clods or milia-like cysts [10,11]. The micronodular subtype of BCC is presented in Figure 2.

#### 3.1.3. Infiltrative BCC

Infiltrative BCC (iBCC) is typically non-pigmented and presents under dermoscopy with arborizing telangiectasia, superficial fine telangiectasia, ulceration/multiple erosions, shiny white-red structureless background and white structureless areas [9,10,11,17,25,26]. Popadić et al. observed that dermoscopic features of iBCC correlated with tumor thickness; thicker areas showed multiple erosions and pigmentation, while thinner areas displayed white structureless zones [15]. Pyne et al. described a dermoscopic “stellate pattern” in iBCC, characterized by geometrical extensions from the tumor margin formed by vessels, surface folds or white linear structures. This feature showed a sensitivity of 31.7% and specificity of 94.1% [27]. “Halo phenomenon” was also reported in a single case of iBCC [28].

#### 3.1.4. Basosquamous BCC

Basosquamous BCC (BSC) shows overlapping dermoscopic features of BCC and invasive SCC. Giacomel et al. suggested that BSC should be considered in the differential diagnosis when at least one dermoscopic finding of both BCC and SCC was present [29]. BCC-related features typically include polymorphous or monomorphous vasculature, while SCC-related findings are linked to keratinization [30]. Polymorphous vascular patterns in BSC consisted of combinations of branched, serpentine, straight, coiled or looped vessels, while monomorphous patterns showed unfocused arborizing vessels [29,30]. Keratinization signs included keratin masses, blood spots on keratin, superficial scales and white clods. Other common features were white structureless areas, shiny white-red background, ulceration and blue-grey blotches [29,30]. Akay et al. introduced two new dermoscopic criteria: “four dots in a square” (rosettes) to differentiate BSC from BCC, and “adherent fibers” (a sign of ulceration) to differentiate BSC from actinic keratosis [30].

#### 3.1.5. Nodular BCC

**Figure 3:**
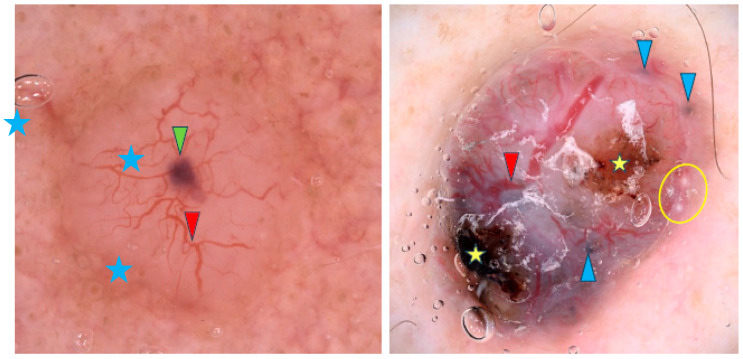
Dermoscopy images of nodular BCCs (nBCCs). The nBCC on the left shows arborizing vessels (red arrowhead), blue clod/ovoid nest (green arrowhead), milky way areas (blue asterisks). The nBCC on the right presents arborizing vessels (red arrowhead), multiple gray-blue globules (blue arrowheads), erosions (yellow asterisks), milia-like cyst (yellow circle).

Arborizing vessels are reported to be characteristic of nodular BCCs (nBCCs), though other vascular patterns like short fine telangiectasias, dots, coils and loops have also been observed [9,11,16,21,31,32,33,34,35,36]. However, one study contradicted these findings and reported no differences in vascular patterns between sBCCs and nBCCs [32]. Translucency is the second most common feature of nBCCs [9,34,36]. Blue-gray ovoid nests are frequently present and, in the study by Popadić et al., were found to have the highest diagnostic accuracy for nBCCs [9,11,13,17,26,36,37]. Other pigmented structures such as blue-gray dots, blue-gray globules and structureless hyperpigmentation are also common [9,21,26,32,36]. Maple-leaf-like structures and spoke-wheel-like areas were identified by Enache et al. as some of the most common dermoscopic findings in pigmented nBCCs [36]. In addition, nBCCs often present with a shiny white-red structureless background or milky red background, though Popadić et al. stated that this finding lacked statistical significance for the nodular variant [9,11]. Among whitish structures, shiny white areas were frequently noted [11,32,36]. Whitish globules may indicate amyloid deposition, as suggested by Park et al. [38]. Interestingly, blue-white veil-like structures and the rainbow pattern were reported more frequently in nBCC than in sBCC [32]. Figure 3 shows examples of nBCCs.

#### 3.1.6. Superficial BCC

Studies are in agreement that short fine teleangiectasias and multiple erosions are significantly associated with superficial BCC (sBCC) [9,11,13,20,25,26,32,33,39,40,41,42,43,44,45].

Some authors have also identified truncated vessels [17] and arborizing microvessels (diameter < 0.2 mm) as typical for this subtype, underlining that arborizing vessels have not been observed [16,21,34,45]. Shiny white to red areas [9,11,16,33,34,35,40,43,44,45], maple-leaf-like areas [9,20,21,25,26,32,37,40,45] and spoke-wheel areas [17,20,32,37,40,46] have also been found as highly specific for sBCC. Other dermoscopic findings frequently observed in sBCC included concentric structures [32], multiple blue-gray globules or dots and ovoid nests [21,26].

**Figure 4:**
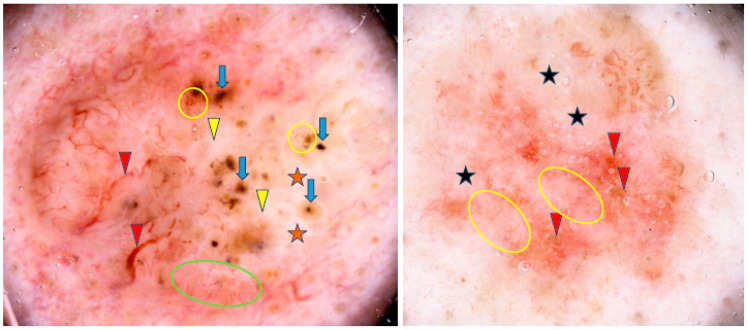
Dermoscopy images of superficial basal cell carcinomas (sBCCs). The sBCC on the left shows arborizing vessels (red arrowheads), shiny white areas/blotches (orange asterisks), concentric structures (blue arrows), short fine teleangiectasias (green circle), shiny white lines (yellow arrowheads), multiple in-focus blue/gray dots (yellow circles). The sBCC on the right presents short fine teleangiectasias (yellow circles), erosions (red arrowheads), milky way areas (black asterisks).

Lallas et al. found that the presence of maple-leaf-like areas and short fine superficial telangiectasias, in association with the absence of arborizing vessels, blue-gray ovoid nests and ulceration, was predictive of sBCC with a sensitivity of 81.9% and a specificity of 81.8% [45]. Some examples of sBCCs are shown in Figure 4.

#### 3.1.7. Fibroepithelioma of Pinkus

Seven articles reported the dermoscopic features of fibroepithelioma of Pinkus (FEP) and were included in the analysis [19,47,48,49,50]. In 2006, Zalaudek et al. analyzed 10 FEPs. All were clinically misdiagnosed as benign lesions but were correctly identified on dermoscopy in 90% of the cases. Key features included fine arborizing vessels, dotted vessels, white streaks and, additionally, gray-brown areas with gray-blue dots in pigmented FEPs (40% of cases). In 2020, Nanda et al. analyzed 48 FEPs, and, apart from serpentine, dotted, or polymorphous vessels and shiny white lines, identified a novel FEP feature—hypopigmented to pink lines intersecting at acute angles (HPLA). This structure has also been reported in hypopigmented melanoma and Spitz nevi [51]. In single case reports, negative network, comedo-like openings and novel findings, including negative maple-leaf-like areas and negative spoke-wheel areas, were reported [47,48,50].

#### 3.1.8. Infundibulocystic BCC

Few cases of infundibulocystic BCC have been reported in the literature, most involving multiple lesions associated with genetic syndromes, with only one case documenting dermoscopic findings. The BCC presented with short fine telangiectasias, maple-leaf-like areas, multiple scattered blue-gray dots and globules, as well as white shiny streaks [52].

#### 3.1.9. Cystic BCC

Cystic BCC is another rare variant of BCC, with only a few reports in the literature, out of which only two included descriptions of dermoscopic findings. Both cases showed arborizing telangiectasia, with one also displaying a homogeneous blue-black area. The blue-black area was suggested to correspond to the cystic regions of the tumor resulting from massive cell necrosis [53].

#### 3.1.10. Blue-White BCC

In one study, the clinically blue-white variant of BCC (n = 32) was analyzed. The authors concluded that the blue color in dermoscopy may result from blue-gray globules, multiple blue-gray dots and/or blue-gray ovoid nests, along with a new dermoscopic finding—homogeneous blue pigmentation, observed in 59% of cases. The homogenous blue pigmentation was suggested to correspond to a large ovoid nest. On the other hand, clinically present white color was linked to dermoscopic structureless white areas, shiny white structures and another novel feature—whitish septa, observed in 44% of BCCs. The blue-white BCCs also frequently presented with ulcerations and fine arborizing vessels [54].

#### 3.1.11. Polypoid BCC

There have been few cases of polypoid BCC reported in the literature, with only three documenting dermoscopic findings. All cases showed arborizing vessels and two also revealed multiple blue-gray globules and ovoid nests [55,56,57].

#### 3.1.12. Large Pore BCC

Lösch et al. described a case of BCC with a central dilated pore, surrounded by a poorly defined whitish-pink area and noticeable branched vessels on dermoscopy. Gray pigmentation and yellowish-white scales were also present around the pore [58].

#### 3.1.13. Linear BCC

The three cases of linear BCCs reported by Alcántara-Reifs et al. were pigmented and showed maple-leaf-like areas and spoke-wheel structures on dermoscopy [59].

#### 3.1.14. Keloidal BCC

A keloid-like portion of BCC displayed arborizing vessels on a pinkish-white background without pigmented components, as reported in one study [60].

#### 3.1.15. BCC with Myoepithelial Differentiation

In BCC with myoepithelial differentiation, dermoscopy revealed irregular linear vessels and arborizing vessels on a whitish background with several dark brown areas [61].

#### 3.1.16. Radiation-Induced BCC

BCCs developing in areas previously treated with radiotherapy showed a predominance of ovoid nests and arborizing vessels on a pink background [62].

#### 3.1.17. Pigmented BCC

Pigmented BCC (pBCC) accounts for less than 10% of BCCs in fair-skinned populations, whereas it represents more than 50%, and even up to 90%, in individuals with skin of color [8,37,63,64,65,66]. However, not all cases of pBCC are clinically evident. Dermoscopy can reveal features of pigmentation in about 30% of clinically non-pigmented BCCs [37,67]. Pigmentation in BCCs results not only from increased melanin but also from a higher number of melanocytes [63,66]. Lallas et al. found that pigmentation is significantly more common in nodular lesions and those located on the trunk [37]. On the other hand, Wolner et al. reported significantly higher incidence of pBCCs on the upper extremities compared with the head and neck region [68].

Park et al. found that increased pigmentation under dermoscopy was associated with a lower likelihood of infiltration and may predict a non-aggressive BCC subtype [63]. This could be linked to the anticarcinogenic properties of melanin, which reduces ultraviolet radiation (UVR) penetration through the epidermis and prevents malignant transformation and tumor cell infiltration. pBCCs were demonstrated to require smaller surgical margins for complete excision, and as a result, they are more often excised with adequate margins. When dermoscopy shows pigmentation within BCC on a level of 11% to 15%, clinicians should anticipate the need for more aggressive surgical treatment [63].

**Figure 5:**
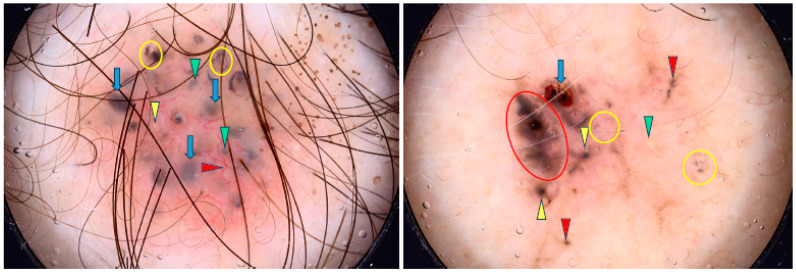
Dermoscopy images of pigmented BCCs (pBCCs). The pBCC on the left shows multiple gray-blue globules (green arrowheads), arborizing vessel (red arrowhead), blue clods/ovoid nests (blue arrows), multiple in-focus blue/gray dots (yellow circles), milia-like cyst (yellow arrowhead). The pBCC on the right presents erosion (blue arrow), maple-leaf-like areas (red circle), multiple gray-blue globules (red arrowheads), multiple in-focus blue/gray dots (yellow circles), milia-like cyst (green arrowhead), spoke-wheel areas (yellow arrowheads).

**Figure 6:**
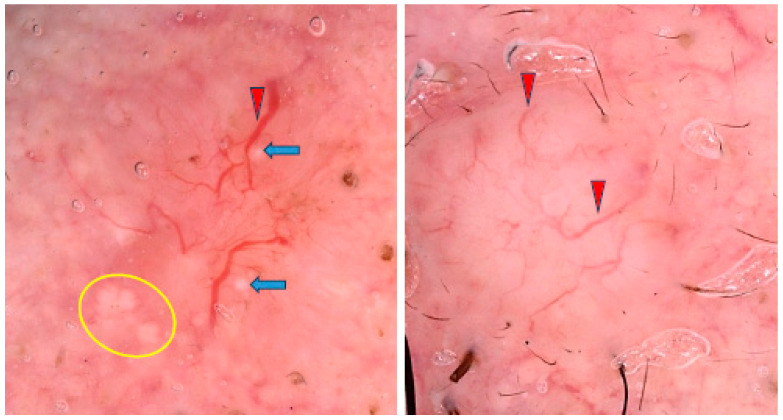
Dermoscopy images of non-pigmented BCCs. The BCC on the left shows MAY globules (yellow circle), milia-like cysts (blue arrows), arborizing vessel (red arrowhead). The BCC on the right presents arborizing vessels (red arrowheads).

Nevertheless, pBCC is not classified as a separate subtype, as pigmented features can occur in all BCC types [37]. Xavier-Júnior et al. distinguished pBCC subtypes with higher-risk morphologies, such as sclerosing or micronodular, and demonstrated that the prognosis is rather related to morphological findings than to the presence of pigmented structures [69]. Examples of pBCCs are shown in Figure 5, whereas non-pigmented variants are illustrated in Figure 6.

#### 3.1.18. Recurrent BCC

BCCs extremely rarely metastasize, and therefore, classical cancer staging systems do not apply to them. Instead, they have been divided based on the risk of local recurrence into two categories—low and high risk.

In the study by Sgouros et al., prognostic features associated with high-risk BCC included clinically apparent white color, the presence of shiny white lines on dermoscopy, nodular morphology and prominent clinico-dermatoscopic ulceration (covering more than 90% of the lesion’s surface). High-risk BCCs more frequently exhibited glomerular vessels and were non-pigmented. Dermatoscopic evidence of pigmentation, on the other hand, was indicative of a low-risk BCC [70].

In another study, a statistically significant correlation was found between arborizing telangiectasia and blue-gray globules with BCC recurrence [55]. Cuellar et al. noted that the first sign of early relapse was the appearance of brown-gray pigmented foci, even when the original tumors were not pigmented [71].

#### 3.1.19. Summary of Dermoscopic Findings by BCC Subtype

In summary, no single dermoscopic finding definitively indicates a specific BCC subtype; however, certain combinations of features may suggest particular subtypes. For instance, the presence of arborizing vessels, translucency and blue-gray ovoid nests may point to nBCC, while short fine telangiectasias, multiple erosions, maple-leaf-like areas, spoke-wheel areas and shiny white-to-red areas are more commonly observed in sBCC. BSC often exhibits a “stellate pattern” and halo phenomenon and sclerodermiform BCC is characterized by arborizing or microvessels on a milky-red, pink-white or porcelain-white background. Table 1 summarizes the dermoscopic characteristics of different BCC subtypes.

Nevertheless, from a clinical perspective, differentiating between “high-risk” and “low-risk” subtypes is more critical than recognizing specific subtypes. High-risk subtypes, including sclerodermiform, micronodular, infiltrative and basosquamous carcinoma, display more aggressive behavior, with greater morbidity and recurrence risk. High-risk BCCs should be suspected in non-pigmented, clinically white lesions that exhibit arborizing, truncated or glomerular vessels; extensive ulceration, especially when covering more than 90% of the lesion’s surface; multiple blue-gray globules; concentric structures and a lack of pink areas or large blue-gray ovoid nests.

### 3.2. Dermoscopic Findings by BCC Location

#### 3.2.1. Face and Scalp

BCC most commonly (up to 80%) develops on the head, particularly on the face and neck, where it typically presents as a nodule with arborizing vessels on dermoscopy [32,33,68,72,73]. In in-depth analysis, Fagotti et al. found that the frontonasal area is the most prevalent location for the nodular subtype, while sclerodermiform BCC is more commonly found in the periauricular area [33]. Facial BCCs, especially in the H-zone (nose, eyes, ears), are more likely to ulcerate and have a higher risk of deeper invasion, indicating more aggressive histological subtypes and a higher risk of recurrence [33,72]. Pogorzelska-Dyrbuś et al. reported a lower prevalence of brown globules in the H-zone and a higher prevalence of glomerular and comma vessels in non-H-zone areas [72,74]. BCCs on the face, particularly in fair and extensively sun-damaged skin, may also appear more subtle, presenting as a white macule or papule. Interestingly, Liopyris et al. observed that 28.9% of these lesions lacked any classic BCC criteria and 13.3% presented with milia-like cysts, making diagnosis particularly challenging. The authors suggested that the white color of the lesion may be attributed to the presence of abundant dermal collagen. Pigmented BCCs are less common on the face [31]. In contrast, scalp BCCs more often display pigmented structures and, surprisingly, also melanocytic patterns, while showing vascular patterns less frequently than BCCs in other locations [32].

#### 3.2.2. Eyelid

In the United States and Western countries, BCC constitutes the most common eyelid malignancy, accounting for up to 90.8% of non-melanoma skin cancers [73,75]. About 20% of BCCs in the head and neck region occur on the eyelids, predominantly on the lower eyelid, especially on the margin, likely due to higher UV exposure [73,75,76,77,78]. Vaccari et al. found that the lateral half of the eyelid margin is most often affected, usually without local symptoms [75]. Pigmented eyelid margin BCCs (EMBCCs) account for 10% of cases, similar to the prevalence in the head region [76]. Madarosis (eyelash loss) is a key sign of EMBCCs and other malignant eyelid tumors [75,76,77].

In dermoscopy, EMBCCs show arborizing, thin linear and polymorphous vessels, as well as linear vessels running perpendicular to the eyelid margin, a feature unique for this location [75,76,77,78]. However, arborizing vessels, while typical for BCC, are not specific to the eyelid [77]. Jaworska et al. highlighted that linear perpendicular vessels are not pathognomonic for EMBCC, as they also occur in normal eyelid margins and other lesions. The authors observed structureless pink areas and starry milia-like cysts in EMBCCs [78]. Williams et al. also reported pink or skin-colored background [77], while Cinotti et al. noted intense pink, yellow and white colors, with yellow possibly due to crusts on erosions [76].

#### 3.2.3. Trunk

In 2022, Jaworska et al. reported that truncal BCCs develop more frequently in younger men. These lesions tend to grow larger and are frequently multiple, likely due to genetic susceptibility and UV-induced oxidative damage to the skin [78]. BCCs on the trunk are mostly superficial and, on dermoscopy, are associated with short fine telangiectasias, spoke-wheel areas and small erosions [32,68].

#### 3.2.4. Areola

BCCs developing on the areola are very uncommon and have been referred to as “BCC of the nipple-areola complex (BCC-NAC)”. Suggested causal factors include UV radiation and radiation therapy, with the latter increasing the risk of multiple BCC-NAC cases, especially when exposure occurs early in life [79]. Kitamura et al. reported a unique dermoscopic finding in BCC-NAC, which they called “large black web”. This feature was characterized by a black network with a mesh thicker than the typical pigment network. Noteworthy is that the pigment network is naturally observed on the areola since it is one of the few naturally pigmented areas of the body [80].

#### 3.2.5. Umbilicus

In 2011, Ramirez et al. reported a case of umbilical BCC in a 21-year-old patient. The small papule was first detected during a full-body mole mapping. Dermoscopy revealed an unpigmented lesion with superficial ulcerations and polymorphic vessels, including arborizing vessels, all suggestive of BCC. The authors underscored that the umbilicus, due to its proximity to various anatomical structures, may facilitate tumor spread. Although only a few cases of umbilical BCC have been documented to date, the case mentioned above is the only one that reported the dermoscopic findings [81].

#### 3.2.6. Limbs

**Figure 7:**
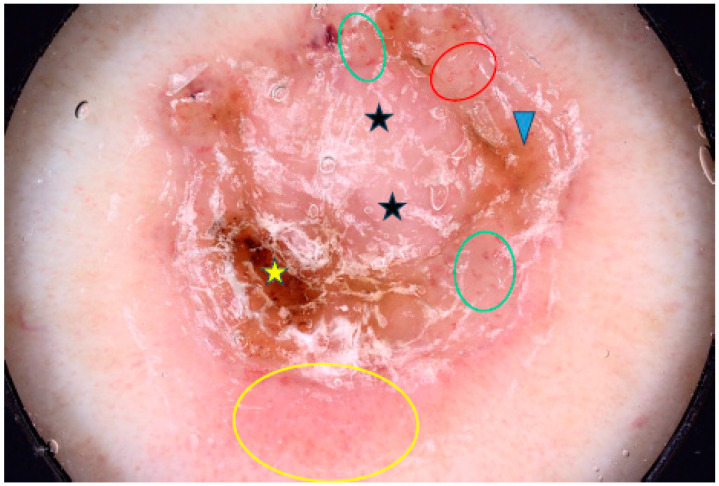
Dermoscopy image of BCC located on the shank showing erosion (yellow asterisk), dotted vessels (yellow circle), looped vessels (red circle), glomerular vessels (green circles), milky way areas (black asterisks), brown homogenous blotch (blue arrowhead).

Four studies on BCCs developing on the limbs were included in our analysis. Wolner et al. evaluated 392 BCCs, of which 54 (13.8%) were located on the lower extremities (LE) and 40 (10.2%) on the upper extremities (UE). BCCs on the LE were more common in women, were diagnosed at a younger age and often showed a superficial subtype. Arborizing vessels were significantly less common on the LE, while ulceration/erosions, polymorphous vessels and shiny white structures were more frequently observed [68]. The BCC located on the LE (shank) is presented in Figure 7.

Two of these studies focused on acral BCCs, which are extremely rare on glabrous skin since BCCs usually develop in hair-bearing areas due to follicular germinative cell differentiation [82]. Factors such as repeated trauma, burns, chronic ulceration, ionizing radiation and arsenic exposure may contribute to their development. Acral BCCs are more common in patients with genetic syndromes such as nevoid basal cell carcinoma syndrome, Bazex syndrome and xeroderma pigmentosum [82,83]. Another explanation is the spread via eccrine ducts [83]. Acral BCCs primarily affect women, though the role of sex hormones is unconfirmed [82]. In dermoscopy, BCCs on glabrous skin present with ulcers, dotted vessels, blue-gray ovoid nests and the absence of arborizing vessels, although one case of periungual BCC did show typical arborizing vessels [82,83,84].

#### 3.2.7. Genitals

UV radiation is considered to be the primary contributing factor of BCC, but it may also develop in non-sun-exposed areas. Advanced age, local trauma, chronic inflammation and radiotherapy may be causative factors in such cases [85]. Studies indicate that vulvar BCC accounts for less than 1–2% of all BCCs and only 2–5% of all vulvar cancers [85,86,87]. Dobrosavljevic Vukojevic et al. reported an even higher prevalence of vulvar BCC, ranging from 2% to 4.9% of all vulvar cancers in Europe, and up to 8% in China [88].

Genital BCCs typically develop on the labium majus and minus of postmenopausal women, with an average age of 70 [85,87,88,89]. Pigmentation in vulvar BCCs is rare in Caucasians (3%) but common in China (81%) [47,86,88]. These cancers are often diagnosed late, usually when the tumor is larger than 1 cm and symptoms like itching, a palpable mass and pain are often misdiagnosed as inflammatory conditions [86,87]. Dermoscopy reveals similar features to cutaneous BCCs, including blue ovoid nests, blue globules, fine telangiectasias, arborizing telangiectasia and white shiny structures, with ulcerations occurring in 28% of cases; however, brown dots have also been described [86,88,89]. In the reviewed literature, no reports of dermoscopic findings for BCC on male genitalia were identified.

#### 3.2.8. Summary of Dermoscopic Findings by BCC Location

In conclusion, BCC most commonly occurs on the head, particularly on the face and neck, where it typically presents as a nodule with arborizing vessels. Non-pigmented BCCs predominate on the face, suggesting a higher likelihood of infiltration and potentially indicating a more aggressive subtype. In the H-zone (nose, eyes, and ears), BCCs are more prone to ulceration, deeper invasion and association with aggressive histological subtypes, leading to an increased risk of recurrence. BCCs in non-H-zone areas more frequently display glomerular and comma vessels rather than arborizing vessels. Approximately 20% of head and neck BCCs occur on the eyelids, with madarosis (eyelash loss) serving as a key sign of EMBCC or other malignant eyelid tumors. On the trunk, BCCs are typically superficial, exhibiting features such as SFT, spoke-wheel areas and small erosions. BCC-NAC should be suspected when a unique dermoscopic feature, described as a “large black web”, is observed. On the lower extremities, diagnosis of BCC is particularly challenging. Lesions in this region more often present with ulceration, polymorphous vessels and SWS, while arborizing vessels are rarely observed. BCC can also occur in non-sun-exposed areas, such as the genital region (vulva) in postmenopausal women, where its dermoscopic features are similar to those seen in cutaneous BCC.

### 3.3. Dermoscopic Features by Age on BCC Onset

A study of 448 BCCs found that early-onset cases (in patients under 50 years of age) were less pigmented and often showed blue-gray globules with no visible vessels. In contrast, arborizing telangiectasia, large blue-gray ovoid nests and ulceration were more common in older patients [90].

### 3.4. Dermoscopic Features by BCC Size

Twelve studies reporting the dermoscopic findings in BCC by tumor size were included in the analysis [21,91,92,93,94,95,96,97,98,99,100,101]. The values used to differentiate between small and large BCCs varied across studies; one study set the cutoff at 15 mm, three at 10 mm, two at 6 mm, two at 5 mm, one at 4 mm, and one at 3 mm [21,91,92,95,96,97,98,99,100,101]. Very small BCCs (vsBCCs) were defined as those measuring 2–3 mm, while micro-BCCs referred to tumors of 2 mm or smaller [21,93].

The included studies demonstrated that the number of established dermoscopic features of BCC increased with tumor size, but no additional size-specific features were observed. However, some dermoscopic findings may be observed with different frequencies, facilitating diagnosis at various stages [91,95,98,100]. Particularly, Ishizaki underscored that dermoscopy contributes to recognizing BCCs in their early stages [96].

Predictors of small BCCs included SFT, small erosions, multiple blue-gray dots and large blue-gray ovoid nests [91,97,98]. On the other hand, some studies led to opposite conclusions. Two of them reported SFT and small erosions to be more frequent in large BCCs [97,99]. Moreover, Wang et al. reported that pigmented structures started to appear at 2 mm, with no difference in frequencies across pBCC sizes [91].

vsBCCs were statistically more likely to present with pigmented structures, particularly blue-gray dots and ovoid nests, but less frequently showed SFT, shiny white structures, ulceration, micro-erosions and scales [21]. The three cases reported thus far of pigmented micro-BCCs demonstrated high dermoscopic variability [93].

In larger BCCs, arborizing vessels, ulceration and SWS are significantly more common [91,94,101]. Arborizing vessels typically appeared when tumors exceeded 6 mm [91]. Kinzel-Maluje et al. concluded that arborizing vessels are the only statistically significant predictor against small BCCs [92].

**Figure 8:**
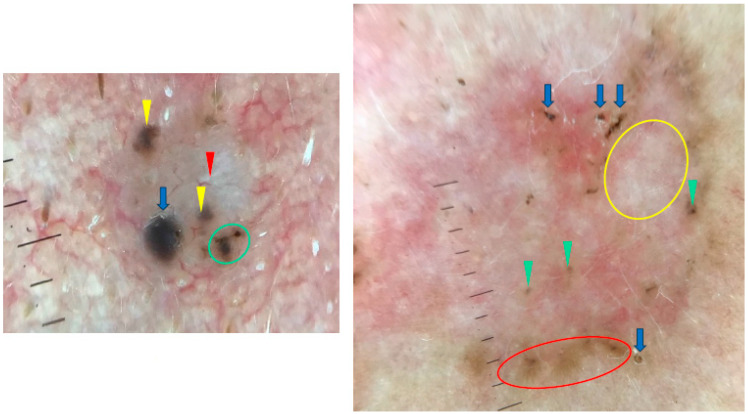
Dermoscopy images of BCCs in different sizes. The small BCC on the left shows blue clod/ovoid nest (blue arrow), multiple gray-blue globules (yellow arrowheads), concentric structures (green circle), shiny white line (red arrowheads). The large BCC on the right presents multiple gray-blue globules (green arrowheads), maple-leaf-like areas (red circle), white areas (yellow circle), multiple erosions (blue arrows).

Arias-Rodriguez et al. demonstrated that aggressive BCC subtypes had similar frequencies of dermoscopic findings regardless of tumor size [21]. Examples of small (3.5 mm diameter) and large (18 mm diameter) BCCs are shown in Figure 8.

Direct comparisons were challenging due to the varying definitions of small and large BCCs used across the publications. Table 2 summarizes findings from the studies included.

### 3.5. Dermoscopic Features by Patient Phototype

We included five studies on dermoscopy in BCC among patients with darker phototypes (III–VI) [66,102,103,104,105] and one study on individuals with albinism [106]. Due to the protective properties of melanin, the incidence of BCC is lower in darker skin tones. The incidence rate of pBCC increases with darker phototypes, from 91.8% in phototypes II-IV to 100% in Black individuals [102,104].

Frequent dermoscopic features included blue-grey dots, ovoid nests, maple-leaf-like areas, blue-white veil, ulceration, arborizing vessels and SFT. Nodular BCC was the most common subtype significantly associated with ulceration, blue-white veil and arborizing vessels [102,103,105]. Maple-leaf-like areas, red-white structureless areas, multiple small erosions and spoke wheel areas were commonly found in sBCC, with the strongest correlation seen for the latter [102,105]. Micronodular BCC showed predominantly arborizing and dotted vessels and blue-white veil [103]. A dermoscopic rainbow pattern was seen in a third of Indian patients [103]. In Black individuals, 55.6% of BCCs had an accentuated reticular network, and 33.3% showed central hypopigmentation, which could be mistaken for a dermatofibroma [104].

In Africans with albinism, BCC was 2.3 times more frequent than SCC, similar to Caucasians, and the most frequent dermoscopic findings included arborizing telangiectasia, ovoid nests and spoke-wheel-like structures [106].

## 4. Discussion

In the current review, we analyzed the variations of dermoscopic features in BCC based on tumor subtype, location, age of onset, size and skin phototype. Data from the literature indicate that there is no single feature that is pathognomonic for a certain BCC subtype or specific to a particular location. This underscores the fact that a combination of dermoscopic features is more relevant for an accurate diagnosis of BCC.

In patients with darker skin phototypes, the incidence of BCC is lower than in lighter skin tones; however, the percentage of pBCCs increases in this group, with blue-grey dots, ovoid nests, maple-leaf-like areas and blue-white veil being frequent findings.

There is also a variety of studies in the English literature on the dermoscopic findings in BCC depending on the tumor size. However, comparing the results between these studies is difficult due to the different ranges of diameter adopted by individual authors. The main attention has been drawn to pigmented structures, which were detected even in 2 mm tumors. Pigmented structures were suggested to be more common in smaller BCCs; however, we presume that their presence rather allows for faster detection of suspicious lesion.

Several factors, beyond the inconsistent definitions of tumor size, may further complicate the interpretation and comparison of results. For example, variability in sample representativeness—such as differences in ethnicity, gender and age distributions—can influence findings. Additionally, the accuracy and reproducibility of dermoscopic assessments, as well as differences in equipment and techniques used in various studies, present further challenges. Addressing these variables is crucial for improving the reliability and generalizability of research on BCC dermoscopy. On the other hand, a limitation of the current review is that only the PubMed database was searched. Another limitation is the lack of consistency in the literature in the division of BCC into clinical and histological subtypes, which causes confusion and makes it difficult to standardize dermoscopic findings.

To address these limitations, future studies should focus on standardizing the definitions of tumor size, improving the diversity and representativeness of samples and establishing protocols for consistent dermoscopic evaluations across populations. Efforts should also aim to develop guidelines for the harmonization of equipment and techniques. Comparative analyses with similar studies could help identify the unique contributions of each work, while also explaining differences or consistencies in findings. This would not only strengthen the understanding of BCC dermoscopy but also enhance its clinical application.

## 5. Conclusions

There is a wide variety of data in the literature on the dermoscopic presentation of BCC depending on the tumor subtype, location, age of onset, size and skin phototype. Despite the evidence confirming the differences in the dermoscopic presentations of BCC, it was not possible to find any pathognomonic feature for any subtype or location.

## Figures and Tables

**Table 1:** Key dermoscopic features of different basal cell carcinoma (BCC) subtypes.

Subtype	Key Dermoscopic Features
Superficial BCC (sBCC)	Spoke-wheel areas, maple-leaf-like areas, multiple erosions, shiny white to red areas, short fine telangiectasias + absence of arborizing vessels, blue-gray ovoid nests and ulceration
Nodular BCC (nBCC)	Arborizing vessels, blue-gray ovoid nests, translucent background, shiny white areas
Basosquamous BCC (BSC)	“Four dots in a square”, adherent fibers, keratin masses
Sclerodermiform BCC	Arborizing vessels on a porcelain-white background, along with multiple erosions or ulcerations (often forming a ring), poorly defined margins
Infiltrative BCC (iBCC)	Stellate pattern, halo phenomenon
Micronodular BCC	Brown and blue globules, white clods, milia-like cysts, arborizing vessels or short fine telenagiectasias, milky red structureless areas, ulceration
Infundibulocystic BCC	Short fine telangiectasias, maple-leaf-like areas, blue-gray dots and globules, shiny white streaks
Cystic BCC	Homogeneous blue-black areas, arborizing telangiectasia
Blue-white BCC	Homogeneous blue pigmentation, whitish septa, blue-gray dots/globules/nests
Polypoid BCC	Arborizing vessels, blue-gray globules, ovoid nests
Large Pore BCC	Central dilated pore surrounded by a poorly defined whitish-pink area, branched vessels, gray pigmentation, yellowish-white scales
Linear BCC	Maple-leaf-like areas, spoke-wheel structures
Keloidal BCC	Arborizing vessels on a pinkish-white background
Fibroepithelioma of Pinkus	Hypopigmented to pink lines intersecting at acute angles (HPLA), fine arborizing, dotted or serpentine vessels, shiny white lines, gray-brown areas, gray-blue dots
BCC with Myoepithelial Differentiation	Irregular linear vessels, arborizing vessels, whitish background, dark brown areas
Radiation-induced BCC	Ovoid nests, arborizing vessels, pink background
Recurrent BCC	Arborizing telangiectasia, blue-gray globules, brown-gray pigmented foci

**Table 2:** Summary of dermoscopic features associated with basal cell carcinoma (BCC) sizes across different studies.

Primary Author, Year, Country	Definitions of BCC Sizes	Dermoscopic Features Predominantly Observed in Specific Sizes
Wang et al., 2024, Taiwan [91]	Small: <6 mm; Large: >6 mm	Small: Short fine telangiectasias, small erosions. Large: Arborizing vessels (appeared in tumors larger than 6 mm), ulcerations, shiny white structures. Pigment patterns appeared at 2 mm and were consistent regardless of size.
Kinzel-Maluje et al., 2024, Brazil [92]	Small: ≤4 mm; Large: >4 mm	Small: Short fine telangiectasia in nodular BCC, concentric structures in micronodular BCC. Large: Arborizing vessels.
Foltz et al., 2023, United States [93]	Micro-BCC: ≤2 mm	Micro-BCC: Dermoscopic variability. The findings include blue-gray dots, blue-gray ovoid nests, spoke-wheel structures, maple-leaf-like structures.
Arias-Rodriguez et al., 2023, Colombia [21]	Very Small: ≤3 mm; Small: 3–10 mm	Very Small: More pigmented structures, including blue-gray dots, globules and ovoid nests, fewer vessels (primarily short fine telangiectasias (SFT)), reduced presence of shiny white structures (SWSs), scales, ulcerations and micro-erosions. Small: Increased number of vessels, more SWS, scales, ulcerations and micro-erosions.
Sykes et al., 2020, New Zealand [94]	Small: ≤41.9 cm^2^ Large: >41.9 cm^2^	Large sBCC: Gain SWS, small blue clods and brown clods over time.
Persechino et al., 2020, Italy [95]	Small: <3 mm	Small pBCCs show typical dermoscopic features of BCC.
Xu et al., 2021, China [101]	Small: <1 cm; Large: ≥1 cm	Large: Blue-gray dots, arborizing vessels, SWS, ulcerations and large blue-gray structureless areas. Large BCCs in heavily pigmented lesions (>70% pigmentation) more frequently exhibited large blue-gray structureless areas, SWS and ulceration.
Ishizaki et al., 2016, Japan [96]	Small: <15 mm; Large: ≥15 mm	Increased detection of smaller BCCs due to dermoscopy; however, no specific dermoscopic findings were evaluated.
Longo et al., 2017, Italy [97]	Small: <5 mm; Large: >5 mm	Small: Multiple blue-gray dots, large blue-gray ovoid nests. Large: Ulceration, multiple small erosions.
Takahashi et al., 2016, Japan [98]	Very Small: ≤3 mm; Small: 4–6 mm	Very Small: Fewer positive dermoscopic features. Both groups: Large blue-gray ovoid nests and multiple blue-gray globules.
Popadić et al., 2015, Serbia [99]	Small: ≤10 mm; Large: >10 mm	Large: Arborizing vessels, short fine telangiectasias, multiple small erosions.
Sanchez-Martin et al., 2012, Spain [100]	Small: ≤5 mm	Small BCCs show typical dermoscopic features of BCC.

## Data Availability

No original datasets were generated for this article.
